# Evolutionary Divergences in Root Exudate Composition among Ecologically-Contrasting *Helianthus* Species

**DOI:** 10.1371/journal.pone.0148280

**Published:** 2016-01-29

**Authors:** Alan W. Bowsher, Rifhat Ali, Scott A. Harding, Chung-Jui Tsai, Lisa A. Donovan

**Affiliations:** 1 Department of Plant Biology, University of Georgia, Athens, Georgia, United States of America; 2 Department of Environmental Health Science, University of Georgia, Athens, Georgia, United States of America; 3 Department of Genetics, University of Georgia, Athens, Georgia, United States of America; 4 Warnell School of Forestry and Natural Resources, University of Georgia, Athens, Georgia, United States of America; North Carolina State University, UNITED STATES

## Abstract

Plant roots exude numerous metabolites into the soil that influence nutrient availability. Although root exudate composition is hypothesized to be under selection in low fertility soils, few studies have tested this hypothesis in a phylogenetic framework. In this study, we examined root exudates of three pairs of *Helianthus* species chosen as phylogenetically-independent contrasts with respect to native soil nutrient availability. Under controlled environmental conditions, seedlings were grown to the three-leaf-pair stage, then transferred to either high or low nutrient treatments. After five days of nutrient treatments, we used gas chromatography-mass spectrometry for analysis of root exudates, and detected 37 metabolites across species. When compared in the high nutrient treatment, species native to low nutrient soils exhibited overall higher exudation than their sister species native to high nutrient soils in all three species pairs, providing support for repeated evolutionary shifts in response to native soil fertility. Species native to low nutrient soils and those native to high nutrient soils responded similarly to low nutrient treatments with increased exudation of organic acids (fumaric, citric, malic acids) and glucose, potentially as a mechanism to enhance nutrition acquisition. However, species native to low nutrient soils also responded to low nutrient treatments with a larger decrease in exudation of amino acids than species native to high nutrient soils in all three species pairs. This indicates that species native to low nutrient soils have evolved a unique sensitivity to changes in nutrient availability for some, but not all, root exudates. Overall, these repeated evolutionary divergences between species native to low nutrient soils and those native to high nutrient soils provide evidence for the adaptive value of root exudation, and its plasticity, in contrasting soil environments.

## Introduction

Mineral nutrient availability in soils is considered a key factor influencing plant productivity and species distributions [1–3]. Although nutrient limitation is common in terrestrial ecosystems, plants have evolved numerous mechanisms to acquire nutrients from soils via their complex root systems. For example, root system morphology, distribution, and architecture play vital roles in exploring the soil for nutrients [4]. In addition to these physical mechanisms for securing nutrient acquisition, plants can chemically influence nutrient availability in soils through root exudation [5,6]. Root exudation, the passive or active release of inorganic ions, volatiles, and primary and secondary metabolites from roots, is a ubiquitous phenomenon in higher plants [7]. Root exudates can increase local nutrient availability in the rhizosphere by influencing soil pH, competing for mineral adsorption sites, chelating mineral nutrients, and solubilizing soil minerals [5,8–11]. Exudates can also indirectly improve plant nutrient acquisition through interactions with microbes. For example, exudation of flavonoids is a critical component for nodule establishment in N-fixing symbioses, and plays an important role in mycorrhizal interactions [12–14]. Root exudates also impact rhizosphere community composition, as well as soil carbon and nutrient cycling [15,16]. Consequently, root exudation is considered to play an important role in plant nutrient acquisition, particularly in low fertility environments [5,17].

Given the potential for root exudation to improve nutrient acquisition by plants, numerous studies have investigated variation among species for root exudate composition [18–20]. Many species respond to nutrient deficiency with increased exudation of organic acids and sugars [21–25], although this response varies both qualitatively and quantitatively among species [22,23,26]. Increased exudation of sugars and organic acids under nutrient deficiency is often expected to be adaptive, given the influence of these metabolites on soil nutrient availability [9,27,28] and microbial community structure [25,29,30]. Therefore, infertile soils may generally favor species with either inherently high exudation of organic acids and sugars, or a high capacity to increase exudation of these metabolites in response to nutrient deficiency [21,23,31]. However, it has also been suggested that exudation of organic compounds in general should be inherently higher in fast-growing species characteristic of fertile soils, due to their high nutrient demand and capacity to sustain rapid metabolic activity via high rates of nutrient assimilation [32,33]. For example, higher exudation in species native to fertile soils may contribute to the faster rates of nutrient cycling observed in the rhizosphere of such species in common garden studies [33,34]. Studies directly comparing species native to low nutrient soils and those native to high nutrient soils are therefore needed to clarify how root exudate composition (i.e. metabolite identity and relative quantity) has differentiated across environments, and to shed light on the role of root exudation in adaptation to soil fertility [35].oweHoHoasdfoweHoHoasdf

Of the studies that have examined variation among species for root exudate composition in relation to native soil characteristics [18,36,37], few have done so in a phylogenetically-informed framework. Controlling for species’ phylogenetic relatedness is an important consideration in comparative studies in order to remove the potential confounding influence of species’ shared evolutionary histories on inferences of adaptive differentiation [38,39]. Similar trait shifts in response to local environmental conditions in multiple phylogenetically-distinct lineages indicates convergent evolution, and provides evidence for the adaptive significance of that trait [40,41]. Thus, while comparative analyses of root exudate composition across species native to contrasting environments are useful for generating testable hypotheses, studies which incorporate species pairs from multiple lineages are particularly informative for making adaptive inferences.

In this controlled environment study, we used gas chromatography-mass spectrometry (GC-MS) to analyze the root exudates of six *Helianthus* species native to habitats differing in soil fertility. GC-MS is best-suited for detection of small, thermostable compounds of primary metabolism, such as sugars and organic acids. Although secondary metabolites such as sesquiterpene lactones have also been detected in root exudates of cultivated sunflower [42], we focused our study on small, primary metabolites which are expected to comprise the majority of root exudates in higher plants [43], and can impact plant nutrient acquisition by influencing both nutrient availability as well as plant-microbe interactions [9,25,27,28–30]. Species were chosen as pairs of phylogenetically-independent contrasts, with each pair including one species native to a relatively low nutrient soil (LNS) and the other native to a relatively high nutrient soil (HNS). Specifically, we asked: (1) Does root exudate composition consistently (i.e. in all three species pairs) differ either quantitatively or qualitatively between species native to LNS and those native to HNS? In addition, responses to changes in nutrient availability (i.e. shifts in root exudate composition between high nutrient-treated and low nutrient-treated plants) may also be adaptive. Therefore, we also asked: (2) Do species native to LNS versus HNS consistently (in all three species pairs) differ in their response to low nutrient treatments? Consistent differences between species native to LNS and their sister species native to HNS in all three species pairs would indicate independent, repeated evolution of root exudate composition (question 1), or its response to low nutrient availability (question 2), providing evidence for its adaptive significance.

## Materials and Methods

### Study system

**Fig 1:**
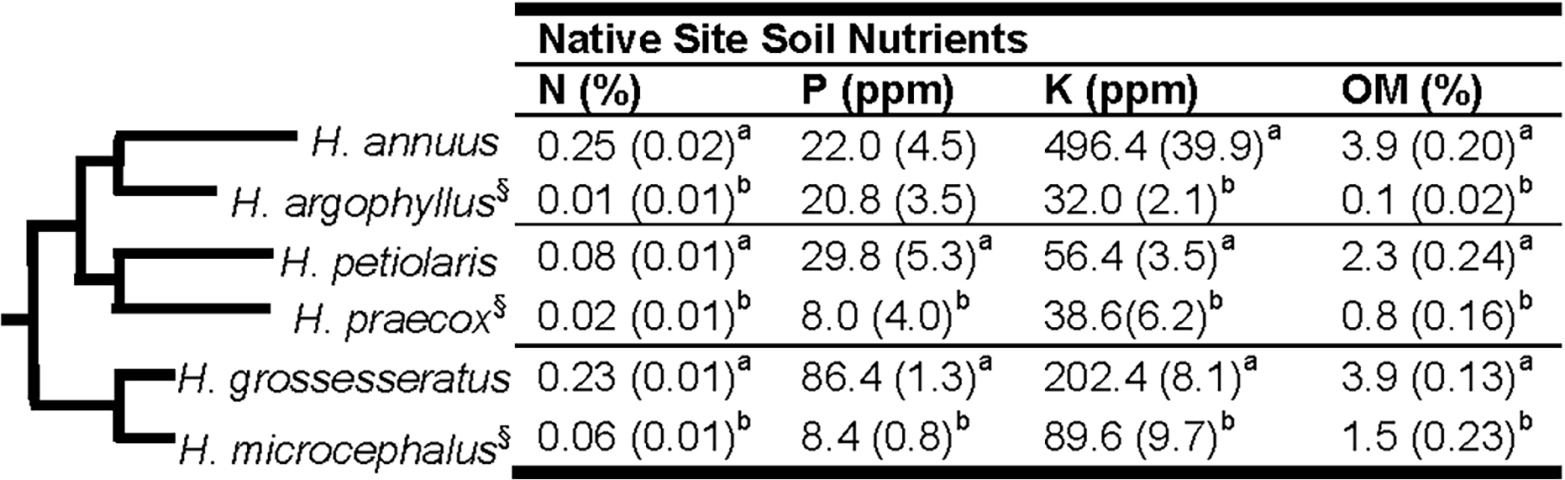
Phylogeny (based on the most recent phylogeny for *Helianthus* [47]) and native soil fertility of the study species. Data are the mean (SE) of five replicates per species. Soil fertility values for four of the species have been previously reported [46]. Different letters indicate significant differences (p < 0.05) between the two sister species within a given clade. Species native to a low nutrient soil (relative to its sister species) indicated by (§). (N) nitrogen; (P) phosphorus; (K) potassium; (OM) organic matter.

The *Helianthus* (sunflower) genus includes approximately 50 herbaceous species found in diverse habitats across North America [44,45]. As part of a separate study, seeds were collected for 28 diploid *Helianthus* species, either directly from wild population native sites, or from accessions established at the USDA National Genetic Resources Program [46]. Soil cores were also collected from the location of each seed source and analyzed for soil fertility characteristics at A & L Laboratories (North Chesterfield, VA, USA), and soil N concentrations were assessed at the University of Georgia Stable Isotope Laboratory (Micro-Dumas combusition; NA1500, Carlo Erba Strumentazione, Milan, Italy). The results of these soil fertility analyses have largely been reported previously [46]. Based on the native soil data for each species, we chose three pairs of sister species (six species total) from different clades in the *Helianthus* phylogeny [47], such that each pair contains one species native to a low nutrient soil (LNS) and the other native to a high nutrient soil (HNS) (Fig 1). Seeds from these six species were all collected directly from wild populations as part of the original seed and soil collections in either 2011 or 2012 at the following locations: *H*. *annuus* (Kansas; N39°06’N 96°36’W), *H*. *argophyllus* (Texas; 27°38’N 97°13’W), *H*. *petiolaris* (Illinois; 41°55’N 90°06’W), *H*. *praecox* ssp. *runyonii* (Texas; 27°39’N 97°18’W), *H*. *grossesseratus* (Illinois; 41°38’N 89°32’W), *H*. *microcephalus* (South Carolina; 34°15’N 82°39’W). Seeds for *H*. *argophyllus*, *H*. *praecox*, *H*. *grossesseratus*, and *H*. *microcephalus* were collected on public roadsides and right-of-ways, and required no permissions for collection. Seeds for *H*. *petiolaris* were collected under a permit from the Illinois Nature Preserve Commission, while *H*. *annuus* seeds were collected with permission from Konza Prairie Biological Station. None of these collections involved endangered or protected species.

### Plant growth conditions, nutrient treatments, and root exudate collection

Seeds were scarified and placed on moist filter paper in petri dishes in the dark for 24 hours before removing the seed coats with forceps. Germinating seedlings were transferred to a growth chamber (Conviron, Winnipeg, Canada) programmed for a 12-hour 25/20°C day/night cycle with 70% relative humidity. Seedlings were grown hydroponically as described [48]. Briefly, one week after scarification, a small section of rock wool was wrapped around the hypocotyl of each seedling, and seedlings were suspended in individual 125 mL Erlenmeyer flasks wrapped in foil. Flasks were filled with 50% strength modified Hoagland’s complete nutrient solution [49] and were aerated using aquarium pumps. Nutrient solutions and rock wool were replaced every other day. Due to seedling mortality in *H*. *microcephalus* and *H*. *praecox*, these two species were replaced with a second set of seedlings germinated 4 weeks after the first set. To assess the potential for confounding temporal effects on root exudation, replicate *H*. *petiolaris* seedlings were germinated in both sets (n = 3 seedlings per set). No significant temporal effects were observed for the abundance of any metabolite detected in *H*. *petiolaris* (t-tests, all p > 0.05); therefore, temporal effects were not considered further.

As root exudate composition is known to vary with plant ontogeny [50], nutrient treatments were initiated when individual plants had reached the same developmental stage to control for differences in growth rates among species. At the emergence of the third true leaf pair, seedling root systems were rinsed with deionized water, then suspended in individual 125 mL Erlenmeyer flasks containing either 50% strength (high) or 1.25% strength (low) modified Hoagland’s complete nutrient solution. For each of the six species, replicate seedlings (n = 5–7 per treatment) were randomly assigned to each nutrient treatment. Nutrient treatment solutions were replaced at two and four days after the initial nutrient treatments. Five days after initiating the nutrient treatments, root exudates were collected as described [48]. Briefly, root systems of intact seedlings were rinsed in deionized water and placed in individual 15 mL glass vials wrapped in foil. Vials were then filled with enough deionized water to cover the root system, and these volumes were recorded. After five hours, the aqueous medium containing root exudates was collected from each vial and adjusted to the original volume. Samples were immediately snap-frozen in liquid nitrogen and stored at -80°C.

Root systems were stained with 0.01% (w/v) Toludine Blue O (Carolina Biological Supply Co, Burlington, NC, USA) for three minutes, then rinsed in deionized water to remove excess stain. Root systems were placed in a clear plastic tray in a thin layer of water to minimize overlap and scanned on a desktop scanner at 400 dpi. Images were analyzed using the software WinRHIZO (v. 2002c, Regent Instruments, Quebec, Canada) for calculation of total root length and surface area. Root systems were then dried at 60°C for 72 hours before weighing. Across species and treatments, both root system length and surface area were highly correlated with root mass (r = 0.831, *P* < 0.0001, n = 75; and r = 0.896; *P* < 0.0001; n = 75, respectively). Therefore, we report root exudate abundance normalized per unit root mass (described below).

### Metabolite analysis

One mL samples of the root exudate collection medium were evaporated to dryness in a vacuum concentrator (CentriVap, Labconco, Kansas City, MO, USA). Samples were then derivatized and analyzed using GC-MS, as described [48,51,52]. Concentrated samples were methoximated with 15 μL methoxyamine hydrochloride (40 mg/mL in pyridine) for 90 minutes at 30°C, then silylated with 30 μL *N*-methyl-*N*-(trimethylsilyl)-trifluoroacetamine for 90 minutes at 60°C. Samples (1 μL) were injected by autosampler onto a DB-5MS column (30 m x 0.25 mm ID, and 0.25 μm film, with deactivated guard column) in 1:5 split mode. An Agilent 7890A GC (Agilent Technologies, Wilmington, DE, USA) was used for metabolite separation using the following temperature settings: 80°C hold for 1 minute, ramping of 20°C min^-1^ to 200°C, then 10°C min^-1^ to 310°C, followed by a 6.5 minute hold. Detection was with an Agilent 5975C quadrupole MS, with source set at 230°C and mass filter set at 150°C. Spectra were collected in scanning ion mode (m/z 50–500) with Chemstation (Agilent Technologies), and, following spectral deconvolution using AnalyzerPro (SpectralWorks, Runcom, UK), peak identities were assigned using both public (NIST08 [53]; Fiehnlib [54]) and in-house MS libraries constructed using authentic standards. Although the use of public databases and in-house libraries allows determination of putative metabolite identities, strict confirmation requires co-elution with pure authentic standards. Future work should confirm the putative metabolite identities reported in this study; however, given that our study detected common, anticipated primary metabolites, we chose a stringent spectral match threshold to improve confidence in assignment of metabolite identities. Metabolite identities were assigned for peaks with a spectral match ≥ 80% in AnalyzerPro (0.7X forward match percentage + 0.3X reverse match percentage) in at least one species and treatment. Peaks were aligned across samples using Metalab [55] as well as manual curation.

Peak areas of each sample were normalized according to the volume of deionized water in which the sample was collected, as well as according to root system dry mass. We then conducted a principal component analysis (PCA) of log-transformed normalized peak areas for visualization of overall differences among species and nutrient treatments in root exudate composition. Missing values comprised a very small portion (less than 4%) of the dataset compared to the typically 10–20% of missing values reported in GC-MS studies [56,57]; therefore, to generate the full dataset required for PCA, missing values were imputed using the mean of the given species and treatment [58–60]. In addition to examining differences in overall root exudate composition by PCA, we also examined whether species native to LNS and their sister species native to HNS differ in exudation of individual metabolites in each of the three separate evolutionary lineages. Therefore, peak areas of each detected metabolite were modeled within each of the three *Helianthus* clades separately, using two-way ANOVA with species, nutrient treatment, and their interaction as explanatory variables. Data were log-transformed as needed to better approximate ANOVA assumptions of normality and homoscedasticity. Effects were considered significant at p < 0.05. Significant effects in ANOVA were extremely congruent when missing data were excluded and when they were replaced with means as described for the PCA; therefore, we report *p*-values calculated with the missing values excluded. All statistical analyses were completed in JMP Pro v. 11 (SAS Institute Inc., Cary, NC).

## Results

**Fig 2:**
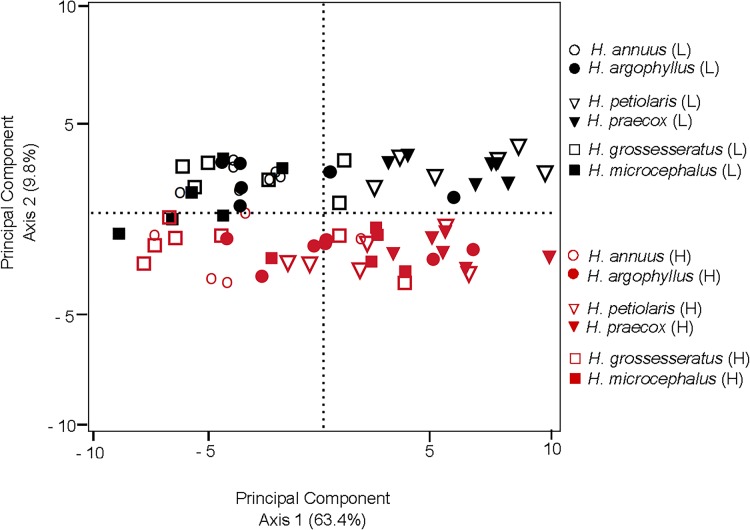
Principal components analysis of abundance of 37 metabolites in root exudates of six *Helianthus* species. Each point represents an individual seedling. Species native to low nutrient soils are filled symbols, while those native to high nutrient soils are open symbols. Seedlings in the low nutrient treatment (L) are black symbols and seedlings in the high nutrient treatment (H) are red symbols.

**Fig 3:**
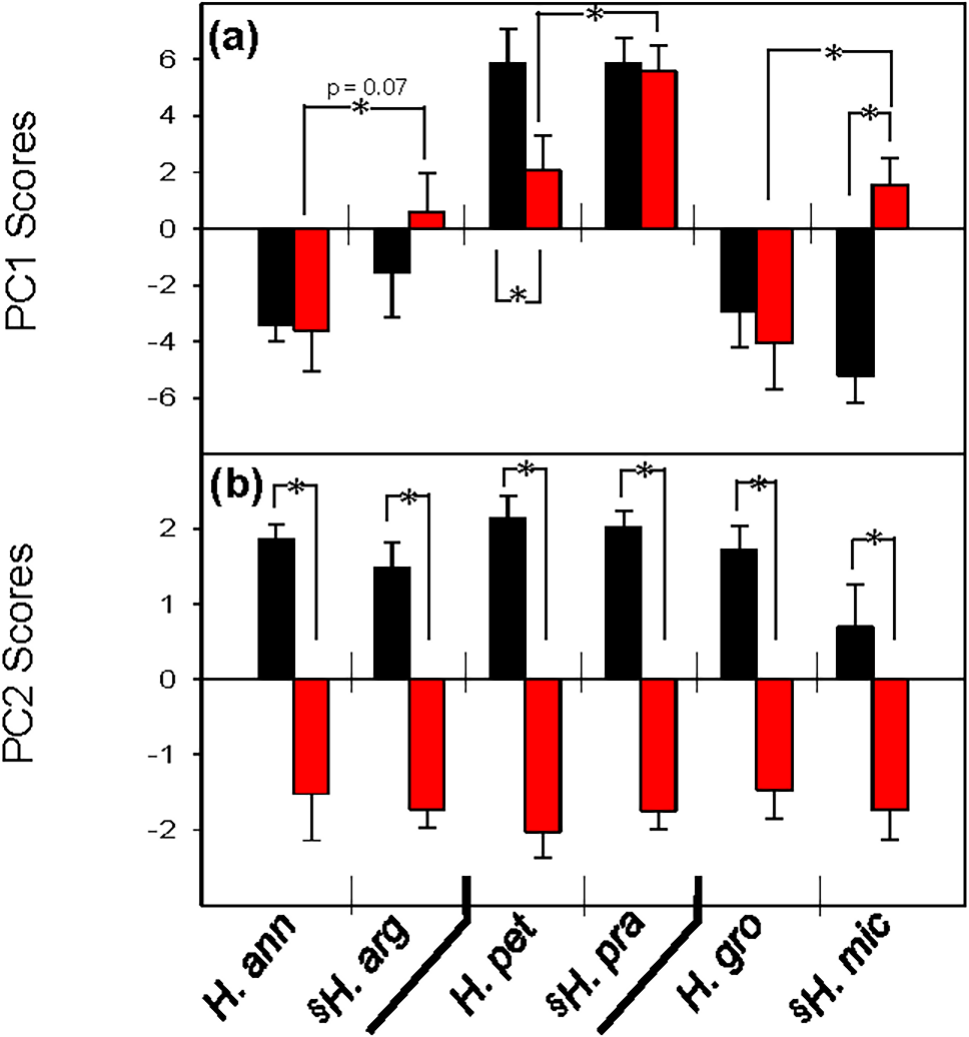
Scores of first (a) and second (b) axes (PC1 and PC2, respectively) of the principal components analysis for root exudate composition of six *Helianthus* species depicted in Fig 3. *H*. *annuus* (*H*. *ann*), *H*. *argophyllus* (*H*. *arg*), *H*. *petiolaris* (*H*. *pet*), *H*. *praecox* (*H*. *pra*), *H*. *grossesseratus* (*H*. *gro*); *H*. *microcephalus* (*H*. *mic*). Data are the mean of 5–7 replicates (± SE) for seedlings treated with either low (black bars) or high (red bars) nutrient treatments. Species are arranged by clade, with the species native to low nutrient soils (relative to its sister species) indicated by (§). Significant differences between species or treatments as assessed by t-tests indicated by (*).

**Fig 4:**
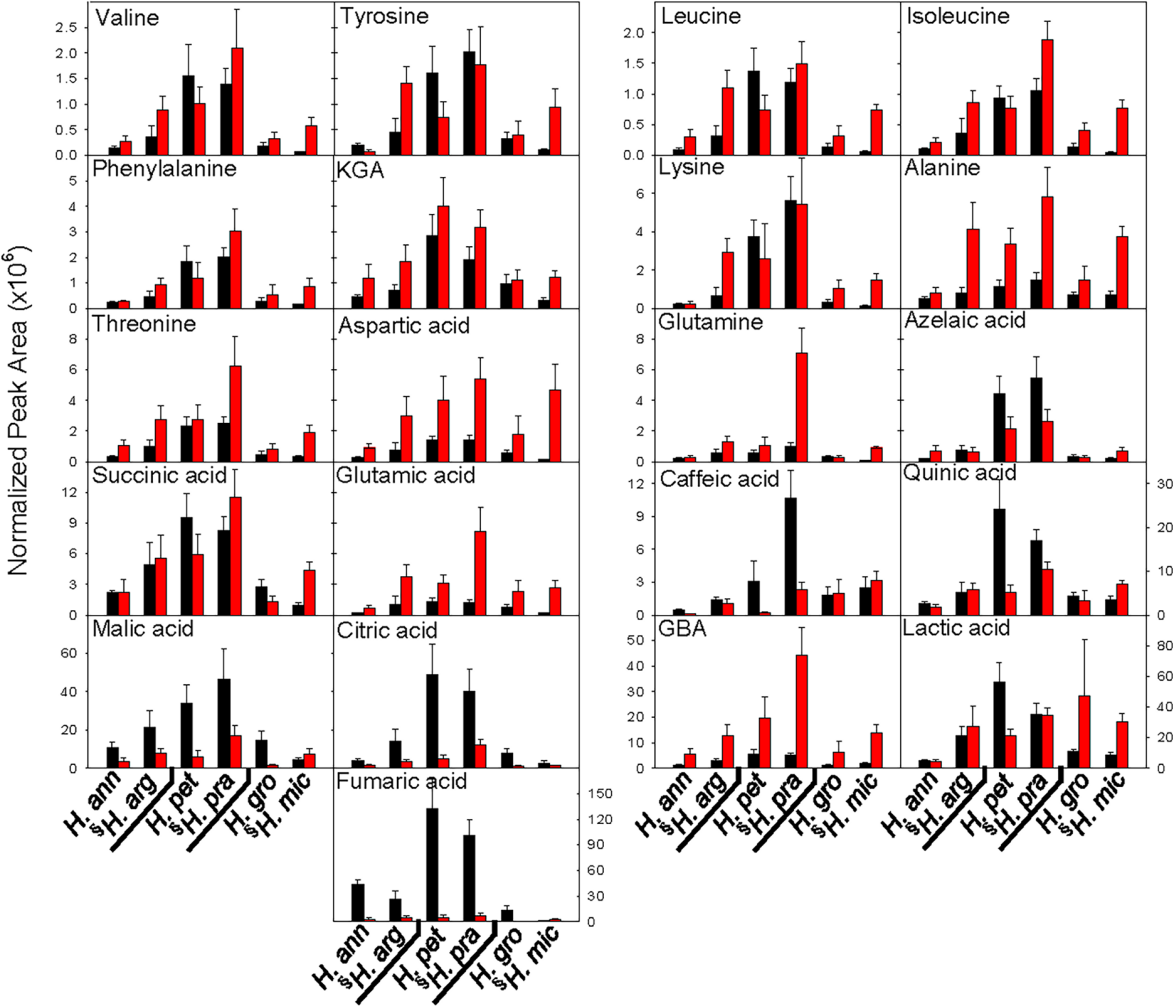
Normalized peak areas of organic acids, amino acids, and derivatives detected under low (black bars) and high (red bars) nutrient treatments in six *Helianthus* species. Data are the mean of 3–7 replicates (± SE) for which those peaks were present. Species are arranged by clade, with the species native to low nutrient soils (relative to its sister species) indicated by (§). γ-guanidobutyric acid (GBA); α-keto-glutaric acid (KGA). Species abbreviations as in Fig 3.

**Fig 5:**
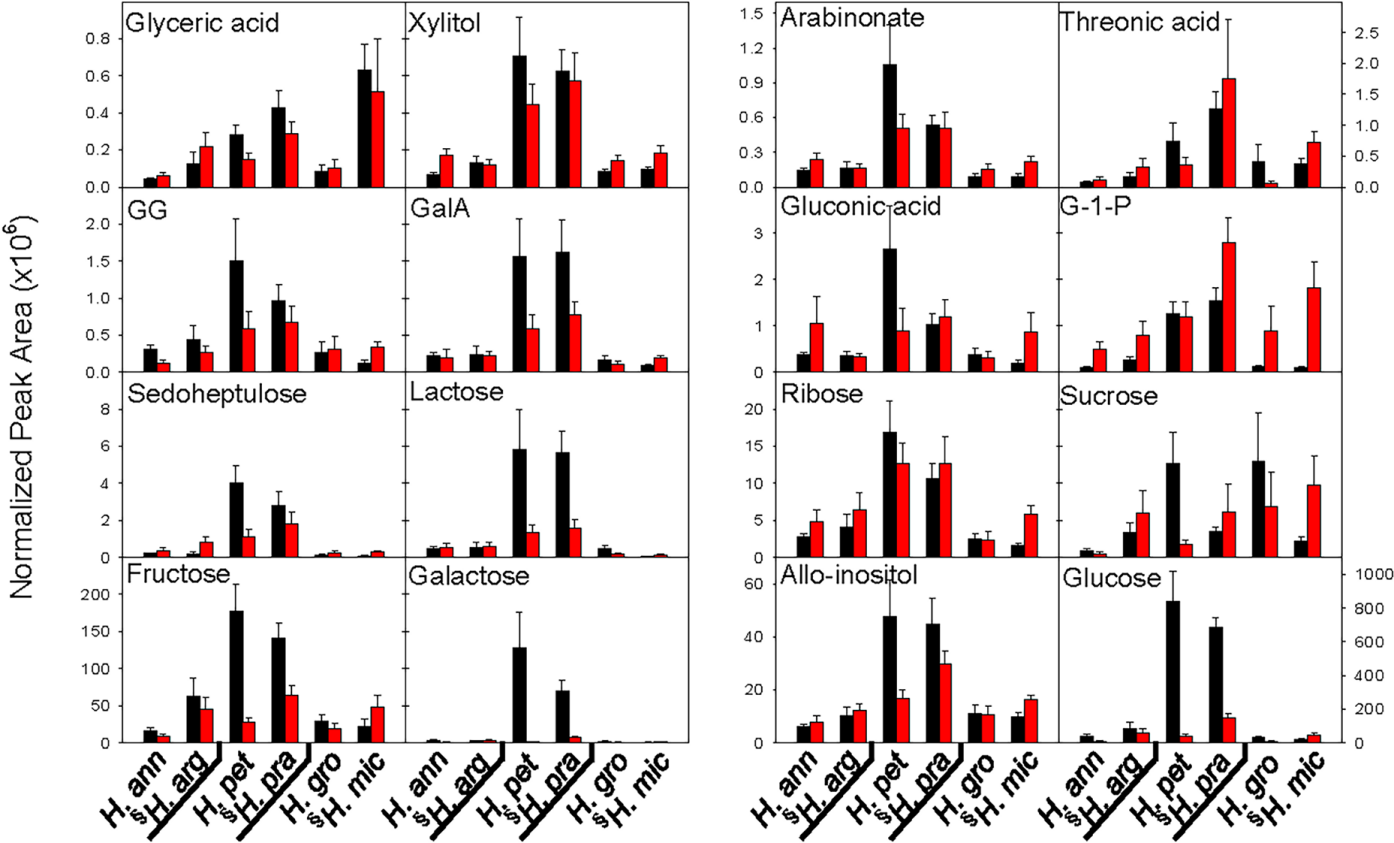
Normalized peak areas of sugars and sugar derivatives detected under low (black bars) and high (red bars) nutrient treatments in six *Helianthus* species. Data are the mean of 3–7 replicates (± SE) for which those peaks were present. Species are arranged by clade, with the species native to low nutrient soils (relative to its sister species) indicated by (§).Glycerol-1-phosphate (G-1-P); galacturonic acid (GalA); 2-O-Glycerol-galactopyranoside (GG). Species abbreviations as in Fig 3.

In total, 37 metabolites were identified across species and treatments, including organic and amino acids, sugars, and sugar derivatives (Table 1). In a principal components analysis of root exudate composition across all individuals, the majority of the variation was accounted for by two principal components (Fig 2). The first principal component (PC1), which accounted for 64.3% of the overall variability among samples, revealed a strong influence of phylogeny, as the *H*. *petiolaris-H*. *praecox* clade (shown in triangle symbols) largely clustered separately from the other two clades along the PC1 axis (Figs 2 and 3A). This can be attributed to an overall higher abundance of most metabolites in the *H*. *petiolaris-H*. *praecox* clade than the other four species (Figs 4 and 5), consistent with the positive loadings of all detected metabolites for PC1 (Table 1).

**Table 1:** Loading scores (± SE) of the detected compounds on the first and second principal components axes (PC1 and PC2, respectively) depicted in Fig 2.

Compound	Loadings	
	PC1	PC2
Alanine	0.69	-0.52
Allo-inositol	0.93	0.08
Arabinonate	0.81	-0.03
Aspartic acid	0.77	-0.47
Azelaic acid	0.91	0.11
Caffeic acid	0.47	0.35
Citric acid	0.73	0.55
Fructose	0.83	0.32
Fumaric acid	0.56	0.62
G-1-P	0.84	-0.31
GalA	0.90	0.20
Galactose	0.82	0.43
GBA	0.72	-0.43
GG	0.83	0.25
Gluconic acid	0.76	0.21
Glucose	0.84	0.44
Glutamic acid	0.73	-0.55
Glutamine	0.73	-0.34
Glyceric acid	0.60	-0.04
Isoleucine	0.83	-0.38
KGA	0.82	-0.21
Lactic acid	0.81	0.03
Lactose	0.84	0.25
Leucine	0.85	-0.29
Lysine	0.79	-0.12
Malic acid	0.65	0.59
Phenylalanine	0.88	-0.12
Quinic acid	0.84	0.20
Ribose	0.92	-0.05
Sedoheptulose	0.87	-0.02
Succinic acid	0.88	0.14
Sucrose	0.46	0.09
Threonic acid	0.80	0.08
Threonine	0.90	-0.36
Tyrosine	0.83	-0.08
Valine	0.90	-0.24
Xylitol	0.81	-0.05

Glycerol-1-phosphate (G-1-P); γ-guanidobutyric acid (GBA); galacturonic acid (GalA); 2-O-Glycerol-galactopyranoside (GG); keto-glutaric acid (KGA).

The clear influence of phylogeny on root exudate composition seen in the principal component analysis (Fig 2) reinforced the need to assess the dataset using phylogenetically-independent contrasts (i.e. comparing root exudate composition within each LNS-HNS species pair) to control for species’ evolutionary relationships. In the high nutrient treatment, the mean PC1 score of each species native to LNS was significantly higher than its sister species native to HNS, although this difference was marginally significant between *H*. *annuus* and *H*. *argophyllus* (p = 0.07; Figs 2 and 3A). This is consistent with the general observation of higher abundance of individual metabolites in species native to LNS than their sister species native to HNS under high nutrient supply (Figs 4 and 5). The results for the high nutrient treatment differed from those for the low nutrient treatment: in the low nutrient treatment, sister species tended to cluster together (Fig 2), and analysis of PC1 scores confirmed that species native to LNS did not significantly differ from their HNS sister species (Fig 3A).

The second principal component (PC2), which accounted for an additional 9.8% of the variance, primarily revealed the influence of nutrient treatments, as indicated by the clear separation between seedlings in the low nutrient treatment (black symbols) and high nutrient treatment (red symbols) on this axis (Figs 2 and 3B). All species exhibited a significantly higher PC2 score under the low nutrient treatment, relative to the high nutrient treatment (Figs 2 and 3B). Organic acids, hexoses, and amino acids were the main drivers of this nutrient response based on PC2 loadings (Table 1). Regardless of species’ native site fertility, low nutrient treatment generally resulted in higher exudation of organic acids (fumaric acid, citric acid, and malic acid) and hexoses (e.g. glucose), and lower exudation of amino acids (glutamic acid, aspartic acid; Tables 1 and 2; Figs 4 and 5) than in the high nutrient treatment. However, significant species by nutrient treatment interaction terms in the ANOVA for 22 of the 37 detected metabolites, including sugars, organic acids, and amino acids, indicated that species native to LNS and those native to HNS responded differently to changes in nutrient supply for some, but not all, metabolites (Table 2). All of those interaction terms were due to relatively larger increases (or smaller decreases in the case of amino acids) in response to low nutrient supply in species native to HNS than their sister species native to LNS (Table 2; Figs 4 and 5). No significant species by nutrient supply interactions were shared across all three clades, indicating that for any given metabolite, species of HNS did not consistently differ from species of LNS in the response to changes in nutrient supply (Table 2; Figs 4 and 5).

**Table 2:** Significant (p < 0.05) p-values of the effects of species (Sp), nutrient treatment (N), and their interaction as assessed by two-way ANOVA on the abundance of root exudates within three different *Helianthus* clades.

Compound	*H*. *ann-H*. *arg*			*H*. *pet-H*. *pra*			*H*. *gro-H*. *mic*		
	Sp	N	Sp*N	Sp	N	Sp*N	Sp	N	Sp*N
Alanine	0.023	-	-	-	<0.012	-	0.049	0.005	0.038
Allo-inositol	-	-	-	-	0.008	-	-	-	-
Arabinonate	-	-	-	-	-	-	-	0.033	-
Aspartic acid	-	0.017	-	-	<0.001	-	-	<0.001	0.004
Azelaic acid	-	-	-	-	0.008	-	-	-	-
Caffeic acid	<0.001	0.006	-	<0.001	<0.001	-	-	-	-
Citric acid	0.029	0.002	-	-	<0.001	-	-	0.006	0.045
Fructose	0.001	-	-	-	<0.001	-	-	-	0.043
Fumaric acid	-	<0.001	-	-	<0.001	-	-	-	0.013
G-1-P	-	<0.001	-	0.021	-	-	-	0.001	-
GalA	-	-	-	-	0.015	-	-	-	-
Galactose	-	-	-	-	<0.001	0.011	-	-	0.042
GBA	-	0.023	-	-	<0.001	-	0.011	0.049	-
GG	-	-	-	-	-	-	-	-	-
Gluconic acid	-	-	-	-	-	-	-	-	0.020
Glucose	0.005	0.005	-	-	<0.001	-	-	-	0.063
Glutamic acid	0.020	0.003	-	-	<0.001	-	-	0.001	-
Glutamine	-	-	-	<0.001	0.009	0.023	-	0.020	0.015
Glyceric acid	0.007	-	-	0.045	0.047	-	<0.001	-	-
Isoleucine	0.008	0.029	-	0.015	-	0.046	-	<0.001	-
KGA	-	0.036	-	-	-	-	-	0.022	-
Lactic acid	0.001	-	-	-	0.042	0.047	-	-	-
Lactose	-	-	-	-	<0.001	-	0.025	-	0.004
Leucine	0.018	0.010	-	-	-	-	-	0.005	-
Lysine	0.004	-	0.029	-	-	-	-	0.029	-
Malic acid	0.046	0.002	-	0.033	<0.001	-	-	0.040	0.022
Phenylalanine	-	-	-	0.037	-	-	-	-	0.034
Quinic acid	0.042	-	-	-	<0.001	0.027	0.036	-	0.011
Ribose	-	-	-	-	-	-	-	-	0.025
Sedoheptulose	-	-	-	-	0.003	-	-	0.045	-
Succinic acid	-	-	-	-	-	-	-	-	0.002
Sucrose	<0.001	-	-	-	0.013	0.035	-	-	0.049
Threonic acid	0.035	-	-	0.008	-	-	<0.001	-	0.006
Threonine	0.044	0.013	-	-	-	-	-	0.009	0.035
Tyrosine	0.001	-	0.004	-	-	-	-	-	0.047
Valine	-	-	-	-	-	-	-	0.029	-
Xylitol	-	-	-	-	-	-	-	-	-

Not significant (-); Glycerol-1-phosphate (G-1-P); γ-guanidobutyric acid (GBA); galacturonic acid (GalA); 2-O-Glycerol-galactopyranoside (GG); keto-glutaric acid (KGA). Species abbreviations are as in Fig 3.

## Discussion

In this controlled hydroponic study, we detected large variation in root exudate composition among six wild *Helianthus* species chosen as phylogenetically-independent contrasts with respect to native soil fertility. Although root exudation has been shown to differ quantitatively between hydroponically-grown plants and plants grown in a solid soil substrate [61–63], differences among genotypes and treatments are widely accepted to be maintained in both hydroponics and soil [18,23,25]. Thus, our objective was to demonstrate relative quantitative differences in root exudation among species and nutrient treatments that are likely maintained in soil, rather than absolute quantities of metabolites which may have little relevance for soil conditions.

Focusing first on the high nutrient treatment, species native to LNS exhibited higher exudation than their sister species native to HNS in all three clades, providing evidence for repeated evolutionary shifts that may be of adaptive significance. Higher overall exudation of sugars, amino acids, and organic acids in LNS species may represent an adaptive strategy for initiating beneficial root-microbe associations, or for accessing relatively immobile nutrients such as phosphorus in the native habitats of these species [64,65]. However, in contrast to the differences between LNS and HNS species within each clade in the high nutrient treatment, no such differences were detected in the low nutrient treatment. Based on similar PCA clustering, no shifts in exudate composition between species native to LNS and their sister taxa native to HNS were observed within any of the three clades, indicating that there is no evidence for consistent differentiation among species under low nutrient supply.

In addition to inherent differences in root exudate composition expressed under a single level of nutrient supply, species responses to nutrient deficiency may also be adaptive. For example, increased exudation of organic acids in response to low nutrient supply has been detected in numerous species [25,27,37,43,48], and has been hypothesized as a mechanism to release mineral nutrients into soil solution through ligand exchange and/or dissolution of soil minerals [9,27,28], while increased exudation of sugars in response to nutrient deficiency may lead to shifts in microbial community structure in the rhizosphere [25,29,30]. Most of the *Helianthus* species generally responded to low nutrient treatments with higher exudation of organic acids (fumaric acid, citric acid, and malic acid) and hexoses (e.g. glucose) than under high nutrient supply (with the exception of *H*. *microcephalus*). The magnitude of this response was similar among species native to LNS and their sister species native to HNS in both of the annual clades, suggesting that the LNS species are not adapted to infertile soils through a unique capacity to increase organic acid and/or sugar exudation in response to nutrient deficiency. Rather, our results suggest that increased exudation of these metabolites is a generally conserved response to nutrient deficiency in *Helianthus*, potentially as a mechanism to improve nutrient acquisition. The only exception was the LNS species *H*. *microcephalus*, which shifted towards lower exudation of nearly all detected metabolites in the low nutrient treatment relative to the high, suggesting that increased exudation of the primary metabolites detected in this study in response to nutrient deficiency is not a mechanism by which this species is adapted to infertile soils.

Although *Helianthus* species generally responded to nutrient deficiency with increased exudation of several organic acids as well as glucose, species native to LNS and their sister species native to HNS differed in their response to nutrient deficiency for a number of other metabolites. For 15 of the 22 metabolites with a significant species by nutrient treatment interaction term in ANOVA, species native to HNS had a larger increase in abundance in response to the low nutrient treatment than their sister species native to LNS. These differential nutrient responses were largely in the *H*. *grossesseratus-H*. *microcephalus* clade, due to *H*. *microcephalus* responding to low nutrients with lower exudation of nearly all metabolites detected in this study. The remaining seven metabolites for which species native to LNS and those native to HNS responded differently to nutrient deficiency (i.e. the remaining seven metabolites with a significant species by nutrient interaction term in ANOVA) were all amino acids. In all three *Helianthus* clades, species native to LNS exhibited a larger decrease in amino acid exudation in response to low nutrient treatments than their sister taxa native to HNS. Decreased exudation of amino acids is a common response to nutrient (particularly nitrogen) deficiency [25,48,66,67], and has been suggested to result from lower amino acid content in roots of nitrogen-deficient plants [25,68]. However, both amino acid influx and efflux are influenced by metabolic inhibitors, suggesting that amino acid exudation results from the combined effects of passive diffusion and active regulation by the plant [69]. For example, amino acid uptake transporters in roots are up-regulated under nitrogen-deficiency [70], resulting in the higher amino acid uptake capacity seen in plants grown under low nitrogen supply [71] and allowing re-uptake of amino acids that were previously exuded into the rhizosphere. The large reductions in net amino acid exudation under nutrient limitation detected in species native to LNS may result from selection to either minimize efflux, maximize influx, or both, for a greater capacity to conserve nitrogen-rich metabolites in LNS.

Taken together, our findings indicate that species native to LNS exhibit higher overall root exudation than species native to HNS under high nutrient supply, and that species native to contrasting soil fertilities exhibit a unique sensitivity to nutrient deficiency for some, but not all, metabolites. Given the unpredictability of nutrient pulses in LNS, there may be selection in LNS for species to maintain generally high exudation rates in order to capitalize during brief periods of high nutrient availability. Such a strategy may help plants of LNS to exploit temporary nutrient pulses, and to prime the rhizosphere for future situations of low nutrient availability by stimulating microbial nutrient cycling [72,73]. Alternatively, high exudation of carbon-rich compounds in LNS may lead to nutrient immobilization, reducing soil nutrient concentrations below that which can be tolerated by fast-growing species with high nutrient requirements [73,74]. Either mechanism is likely to improve plant fitness in LNS, resulting in selection for high exudation rates. In contrast, the lower root exudation in species native to HNS under high nutrient supply may suggest that these species have adapted to their environment by mechanisms other than root exudation. Species native to chronically HNS may rely on their generally fast root growth rates to a greater extent than root exudation in order to rapidly exploit nutrients and gain an edge over competitors in high nutrient conditions [75,76]. In response to nutrient deficiency, however, the increased exudation of organic acids and hexoses detected for HNS species (as with LNS species) may be a means for chemically or biologically (i.e. microbial mining) increasing nutrient acquisition [73,77]. Given that root tissues are expected to be longer-lived in species native to LNS, the rapid root growth strategy is likely not viable in LNS, even in nutrient-rich soil patches, as rapid production of new roots may be too expensive to maintain as those patches are depleted [78–80]. Species native to such LNS, rather, are generally expected to grow slowly, but maintain long-lived root systems which can tolerate extended periods of low nutrient availability [78–79]. For plants native to LNS, root exudation may therefore represent a more cost-effective strategy for maximizing nutrient acquisition than allocating resources for increased root length production.
